# Digital-twin driven alignment control method for marine shafting with air spring vibration isolation system

**DOI:** 10.1038/s41598-025-85196-8

**Published:** 2025-01-07

**Authors:** Song Liu, Liang Shi, Wei Xu, ZeChao Hu

**Affiliations:** 1 Naval University of Engineering, Wuhan, Hubei China; 2 National Key Laboratory on Ship Vibration & Noise, Wuhan, China

**Keywords:** Alignment control, Digital twin, Air spring vibration isolation system (ASVIS), Marine shafting, Proportional-integral-derivative (PID), Computer science, Electrical and electronic engineering

## Abstract

Shafting alignment is crucial for marine propulsion systems and may affect the safety and stability of ship operations. Air spring vibration isolation systems (ASVISs) for marine shafting can help control the shafting alignment state by actively adjusting air spring pressures while effectively reducing the mechanical noise. However, how to accurately control the alignment state of marine shafting with air spring vibration isolation system remains a challenge. To address this issue, a digital twin (DT)-driven alignment control method is proposed in this paper. First, we design a digital twin prediction model based on the neural network to describe the data mapping relationship between the air spring pressures and shafting alignment state. Then, based on the prediction model, we transform the shafting alignment control problem into a non-linear optimization problem in which our objective is to minimize the alignment error while balancing the load on different air springs. To obtain the optimal air spring pressures, the genetic algorithm is introduced to solve the optimization problem, fully exploiting its global search capacity. Moreover, in order to achieve the optimized pressures, a soft-constrained controller based on proportional-integral-derivative (PID) algorithm is developed to accurately generate specific control policies based on the monitoring data. Finally, the feasibility and the effectiveness of the proposed alignment control method is verified with a real ASVIS.

## Introduction

In recent years, air spring vibration isolation systems (ASVISs) have been widely applied in marine propulsion systems to reduce mechanical noise caused by rotating machinery and other related equipment^1–4^. But when there are external disturbances such as working condition change, hull deformation and structure aging on the marine, the resilient support of the vibration isolation systems may cause shafting misalignment. Poor shafting alignment state not only generates additional abnormal noise, but also lead to faults such as shaft fatigue cracks, damaged bearing, which may endanger the safety and the stability of ship operations^5^. Fortunately, air spring vibration isolation systems can help control the shafting alignment state for marine propulsion system and keep the driving shaft and driven shaft in the same center line, by monitoring the system status in real time and actively adjusting the air spring pressures. However, for large scale ASVISs, due to factors such as raft deformation, hull deformation and complex working conditions, there are difficulties in modeling and developing control polices for shafting alignment, making accurate alignment control still a challenge.

In order to control the alignment state, we need to first obtain and model the accurate alignment state of the marine shafting. In the engineering, four components are commonly used to represent the alignment state, namely vertical offset, horizontal offset, vertical angularity and horizontal angularity. And for good alignment state, the vertical/horizontal offsets should be less than 0.5mm and the vertical/horizontal angularities should be less than 0.5mm/m^6^. And there are mainly two methods to get the alignment state: direct measurement method and indirect measurement method. For indirect measurement methods, abnormal signals such as vibration and current can be generated when the shaft system is misaligned^7,8^. By collecting these signals, it is possible to determine whether the shaft system is aligned, but this method cannot accurately describe the alignment state. For direct measurement methods, eddy current sensors or laser sensors are usually used to measure the alignment state. Shi et al.^4^ use eddy current sensors to measure the displacement height difference at different positions, and then calculate the alignment state using a specific mathematical formula. Garg et al.^9^ use laser sensors to directly measure the alignment state. Cheng et al.^10^ proposed a hybrid method to solve the monitoring of the alignment state under the deformation condition of the hull. These methods can measure and calculate the alignment state, but they do not establish a connection between the control objective (alignment state) and the control object (air spring pressure), which cannot help make control decisions directly.

The second issue is how to dynamically control the shafting alignment. Scholars have conducted extensive research on shaft alignment control. Most of these studies focused on the static shafting alignment issues during the design and installation phases^11–14^. For example, Ho et al.^14^ designed an intelligent hybrid Taguchi-genetic algorithm to optimize bearing offset and shaft alignment, in which bearing stress, shear force and permissible reaction force under cold and hot operating conditions are considered. For the dynamic alignment control during the operation phase, it is mainly achieved through adjusting the air spring pressure of the ASVIS in engineering. He et al.^15^ applied the ASVIS to ship propulsion system, analyzed the influence of main design parameters on system stability and natural frequency, proposed convergence criteria for alignment control and safety protection mechanism under extreme conditions. Lv et al.^16^ discussed the feasibility of attitude control by adjusting air spring pressures for the three-phase dynamic balance control problem of floating raft isolation devices, and analyzed the various disturbance force characteristics of raft attitude changes. Bu et al.^17^ proposed a multi-objective coordinated attitude control method for dual-layer air spring vibration isolation mounting, which can adapt to the attitude control coupling of the superstratum and the substratum air spring isolation mounting. Shi et al.^4^ analyzed the mechanical characteristics of variable mass floating rafts and proposed a control method that can adapt to the changes in liquid tank mass from full load to empty load, maintaining high attitude control accuracy. These methods are mainly designed based on the ideal control response model, which is mathematically derived to describe the relationship between air pressure changes and alignment/attitude state changes. However, due to the difficulty in obtaining accurate parameters such as the gravity and mass of the device required to establish a control response model, skilled engineers are usually needed to adjust the parameters and specific control policies on site. And these rule-based control methods are hard to adapt to environmental changes, which may pose a risk of reduced control accuracy during the long-term operation of the system. Therefore, it is necessary to explore more effective online alignment control methods for shaft systems.

The digital-twin technology provides a new approach for shafting alignment control. Digital-twin technology is achieved by establishing virtual twin models of physical entities, updating them based on real-time monitoring data, and utilizing simulation, machine learning, and inference to assist decision-making. This technology has been widely applied in fields such as fault diagnosis and lifecycle management. Zi et al.^18^ built a digital-twin for milling tool wear monitoring system for real-time tracking of milling cutter wear in the manufacturing process, improving the accuracy of milling cutter wear status prediction. Pan et al.^19^ proposed a data-driven model predictive control approach for robotic penguin depth control, maximizing the benefits of the controller while ensuring safety and stability. Min et al.^20^ designed a smoke alarm calibration system based on digital-twin technology, which increased the qualified rate and reduced the repair rate of defective products. Unfortunately, we have not found any relevant research on the application of digital twin technology in shaft alignment control.

In this paper, we proposed a digital twin-driven alignment control method for marine shafting with ASVIS, leveraging real-time data to model and control the system. Firstly, we estabilish a digital twin prediction model for the ASVIS physical entity to describe the data mapping relationship between alignment state and air spring pressures and the back-propagate neural network (BP-NN) is used as the surrogate modeling method. The digtal twin prediction model can be updated in real-time through monitoring system data. Then, based on the prediction model, the shafting alignment control problem is transformed into a multi-objective non-linear optimization problem, in which the objective is to minimize the alignment error and balance the load on different air springs. In order to obtain the air spring pressures with optimal shafting alignment state, a heuristic optimization method based on genetic algorithm (GA) is designed. By employing this method, a group of air spring pressures with the optimal alignment state for the ASVIS can be calculated. Furthermore, in order to accurately control the system according to the optimized air spring pressures, a soft constrained proportional-integral-derivative (PID) controller is developed. The experimental results show that the proposed digital twin-driven shafting alignment control method can accurately and effectively control the shaft alignment state.

The main contributions of this paper are as follows:

- To the best of our knowledge, this is the first study to integrate digital twin technology with marine shafting alignment control and propose a digital twin-driven alignment control framework to solve the alignment control problem. And the shaft alignment control problem is decomposed into a state optimization problem based on the prediction model and a state control problem in the ASVIS.
- The state optimization problem based on prediction model is theoretically formulated as a multi-objective non-linear optimization problem, in which the objective is to minimize the alignment error while balance the load on different air springs. In order to obtain the optimal air springs pressures, we develop a heuristic algorithm based on the genetic algorithm.
- In order to precisely control the system according to the optimized air spring pressures, we design a soft constrained PID controller. The controller can convert the difference between the current air spring pressures and the optimal pressures into solenoid valve control time, achieving fast and accurate pressure control.
- Extensive experiments have been conducted to verified the feasibility and effectiveness of the proposed method. The results show that the digital twin driven alignment control method can accurately control the alignment state based on optimized pressures while maintaining load balancing.

## System model and problem description

### System model

**Fig. 1:**
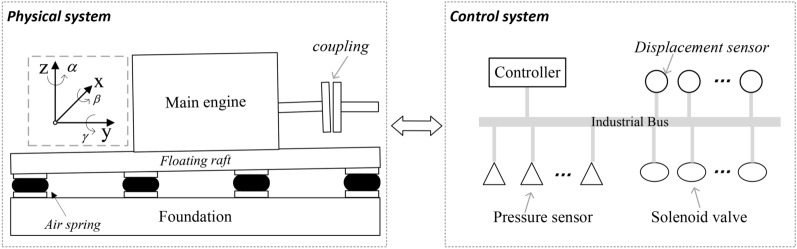
the schematic diagram of an air spring vibration isolation system and its coordination system.

An air spring vibration isolation system usually consists of a set of air spring isolators and a control system. The air springs can isolate mechanical noise by directly supporting equipment or indirectly supporting equipment with a floating raft. And when the ship’s shaft system deviates or deflects, the support height of the air spring can be changed by actively adjusting the distribution of air spring pressure, thereby adjusting the alignment state of the shaft system. For the convenience of description, we establish a coordinate system as shown in Figure 1. And we set the output shaft of the main engine as the positive direction of the *y*-axis, the vertical upward direction is the *z*-axis, and the horizontal direction is the *x*-axis. In engineering, the alignment state of the marine shafting is generally described by four components, that is, vertical offset $x_c$, horizontal offset $z_c$, vertical angularity $\alpha$, horizontal angularity $\beta$. The units of vertical/horizontal offset and vertical/horizontal angularities are usually expressed in *mm* and *mm*/*m*, respectively. Here, the unit *mm*/*m* (millimeter/meter) represents the amount of angle change per meter of length, which is actually a dimensionless measurement used to describe the magnitude of angle change. Especially within a small angle range, the angle value and slope value are almost equal, so *mm*/*m* can be regarded as the unit of measurement for angle. For example, 0.005*mm*/*m* is approximately equal to $5 \times 10^{-6}$ radians. The ideal alignment state is usually considered as the origin state and we set it to zero. Then, the alignment state vector in the coupling center point can be expressed as:

1 $$\begin{aligned} \Gamma = {\tau _1, \tau _2, \tau _3, \tau _4} =\{x_c, z_c, \alpha , \beta \} \end{aligned}$$

The control system of the ASVIS typically consist of pressure sensors, solenoid valves, displacement sensors, and a controller. The pressure sensors are used to measure air springs’ internal gas pressure. Solenoid valves are used to control the inflation and deflation of air springs. Displacement sensors are used to measure the height difference between the foundation and the equipment, which can be used to calculate the alignment status of the shaft system. The air springs’ pressures and displacement sensor data are transmitted to the controller through the industrial bus. The controller can complete computing tasks such as model training, and control strategy generation. And it can also control the solenoid valve through the industrial bus to inflate or deflate an air spring.

### Problem description

To maintain a good alignment state, two aspects should be considered: 1) alignment precision; 2) load balancing. Firstly, in the process of adjusting the alignment state, we hope it converges to the ideal alignment state as much as possible, and each component in the alignment state vector should meet the alignment requirements. In other words, we need to minimize the distance from the current alignment state $\Gamma$ to the ideal alignment state:

2 $$\begin{aligned}&\text {min }{{d}_{1}}(S)=\big | \Gamma \big |=\sqrt{{{x}_{c}}^{2}+{{z}_{c}}^{2}+{{\alpha }^{2}}+{{\beta }^{2}}} \nonumber \\&\text {s}\text {.t}\text {. }\tau _{i}^{l}\le {{\tau }_{i}}\le \tau _{i}^{u},{{\tau }_{i}}\in \{{{x}_{c}},{{z}_{c}},\alpha ,\beta \} \end{aligned}$$

Where $\tau ^u_i$ means the upper bound of and $\tau ^l_i$ means the lower bound of $\tau _i$. For example, in the engineering, when the vertical/horizontal offset is less than 0.5*mm* and the vertical/horizontal angularity is less than 0.5*mm*/*m*, the marine shafting system is considered in good alignment state. Then the upper and lower bound of the alignment state component $\tau _i$ can be set to $\tau ^u_i = 0.5$ and $\tau ^l_i = -0.5$, separately. *S* represents the system state of an ASVIS, mainly including air spring pressures and operating condition. Thus, it can be also written as $S=\{P,C\}$. $P=\{p_i | 1\le i \le N_P\}$, and $p_i$ represents the *i*-th air spring pressure. $C=\{c_j | 1\le j \le N_C\}$ , $c_j$ represents the operating condition of the system, such as roll angle, pitch angle, etc.

Secondly, in the design of an ASVIS, it is usually desirable to evenly distribute the weight of the supporting equipment as much as possible, that is, to maintain load balance. Considering that the pressure of the air spring is approximately linearly related to its load, and the pressure is easy to be measured and analyzed, we use the pressures as the control variable. To achieve load balancing, the mean square error of air spring pressure should be minimized:

3 $$\begin{aligned}&\text {min }{{d}_{2}}(S)=\sqrt{\frac{1}{N}\sum \limits _{i=1}^{N}{{{({{p}_{i}}-\overset{\_}{\mathop {p}}\,)}^{2}}}} \\ \nonumber&\text {s}\text {.t}\text {. }p_{i}^{l}\le {{p}_{i}}\le p_{i}^{u},i\in [1,N] \end{aligned}$$

where ${\bar{p}} = \frac{1}{N} \sum \nolimits _{i=1}^{N}{{p_i}}$ is the average pressure of *N* air springs, $p_i^u$ means the upper bound of $p_i$ and $p_i^l$ means the lower bound of $p_i$. In an ASVIS, air springs should operate within the safe pressure range. For example, if all air springs are the same and the safe pressure range is $0\sim 2.0$Mpa, then $p_i^l=0$ and $p_i^u=2.0$.

To minimize the alignment control error while maintain load balancing, the shaft alignment control problem can be formulated as a non-linear optimization problem:

4 $$\begin{aligned} \text {min }d(S)= & {{d}_{1}}(S)+\xi \centerdot {{d}_{2}}(S)=\sqrt{{{x}_{c}}^{2}+{{z}_{c}}^{2}+{{\alpha }^{2}}+{{\beta }^{2}}}+\xi \centerdot \sqrt{\sum \limits _{i=1}^{N}{{{({{p}_{i}}-\overset{\_}{\mathop {p}}\,)}^{2}}}} \\ \nonumber= & \big | f(S) \big |+\xi \centerdot \sqrt{\sum \limits _{i=1}^{N}{{{({{p}_{i}}-\overset{\_}{\mathop {p}}\,)}^{2}}}} \\ \nonumber \text {s.t. } & \tau _i^{l}\le {{\tau }_{i}}\le \tau _{i}^{u},{\tau _i}\in \{x_c,z_c,\alpha ,\beta \} \\ \nonumber & p_{i}^{l}\le {{p}_{i}}\le p_{i}^{u},i\in [1,N] \end{aligned}$$

where $\xi$ is a constant. And considering that our control object is the air spring pressure, we use a function to describe the relationship between the air spring pressures and the alignment state, denoted by:

5 $$\begin{aligned} \Gamma = f(S) = f(P,C) \end{aligned}$$

However, it is difficult to obtain the mapping relationship between the system state and the alignment state through mathematical modeling and empirical formulas. Therefore, in this paper, we construct a digital twin model to describe the data mapping relationship between *S* and $\Gamma$, and update the model in real-time by monitoring the system data. The details will be introduced in the next Section.

## Digital-twin driven alignment control method

### System framework

**Fig. 2:**
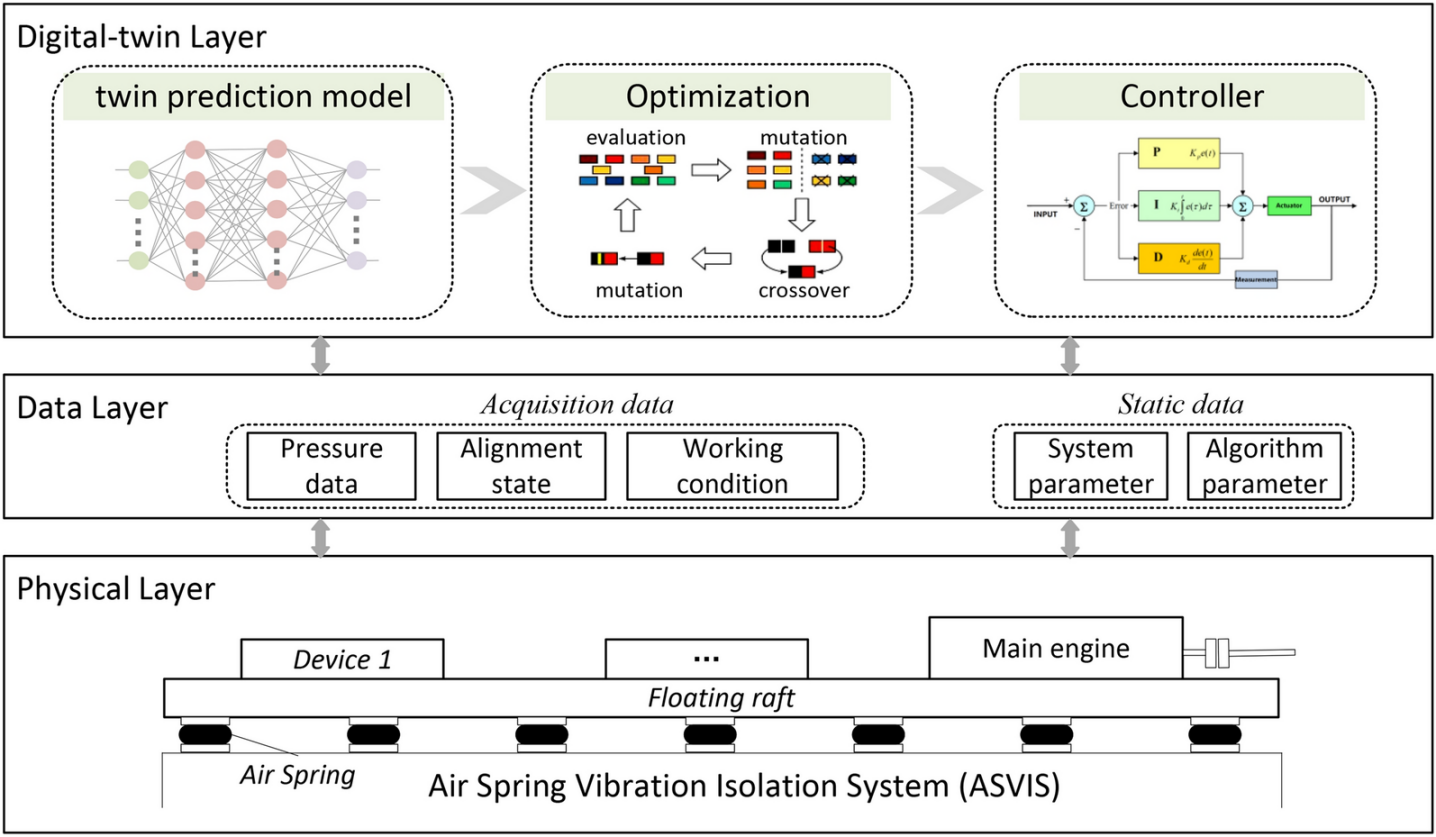
The system framework of digital-twin alignment control method.

The system framework of digital twin-driven alignment control method can be divided into three logical layers: physical layer, data layer and digital-twin layer, as shown in Figure 2. The physical layer mainly includes ASVISs, load-bearing equipment, etc., which are real physical entities. In the data layer, there are two kinds of data: time-varying data and static data. The time-varying data is collected by the control system of ASVIS, including pressure data, alignment state and working condition. Static data mainly includes the parameters used by the system and algorithm, which are generally set by administrators. In the digital-twin layer, a twin prediction model is first established and continuously updated according to the information in the data layer to describe the data mapping relationship between the system state and alignment state. Then, a heuristic algorithm based on the genetic algorithm is developed to solve the alignment control problem as shown in Equation 4, and a group of optimal air spring pressures will be obtained. According to the optimized air spring pressures, the controller can generate specific control policies and execute them.

During the control process, the system state and alignment state will be changed by executing control strategies, and the controller will monitor the state in real-time to achieve precise control. In addition, as the physical entity state changes, the twin prediction model in the digital-twin layer will also be updated based on the time-varying data in the data layer. After the prediction model is updated, the optimization algorithm will recalculate the optimal pressures and hand it over to the controller for execution. While existing research has primarily focused on static alignment control or rule-based dynamic adjustments, we introduce this digital twin-driven method, combining real-time system updates, global optimization, and adaptive control. We believe that this methodology can improve the performance in precision and adaptability over conventional methods.

### Digital-twin prediction model

To optimize the alignment state, we need first establish a digital-twin model to describe the relationship between the system state and the alignment state. Due to the fact that the ASVIS is a complex nonlinear system, it is difficult to use a theoretical model to describe the relationship between air spring pressures and the alignment state. The BP-NN is known for their ability to model complex, nonlinear relationships, and it can capture the intricate dynamics of the ASVIS without explicit modeling. The BP-NN model can also adapt to changes in the system dynamics by learning from new data, making it inherently more robust to variations, such as those caused by system aging. Thus, BP-NN is used as the surrogate modeling method. In this subsection, the details of the digital-twin prediction model based on BP-NN will be presented. A BP-NN generally consists of an input layer, several hidden layers and an output layer. And each layer has plentiful neurons to imitate the biological nervous system.

The system state $S=\{s_1,...,s_N\}$ is the input vector for the input layer. As mentioned above, the system state includes air spring pressures *P* and the working condition *C*. Thus, $N=N_P+N_C$. On the other hand, for specific working condition, the alignment state is only affected by air spring pressures. The output vector is the alignment state $\Gamma$, so that the nodes number in the output layer is $M=4$. The hidden layer is usually set according to the complexity of the system. In this paper, we uses a single hidden layer network, the number of hidden layer nodes can be calculated by following empirical formula^21^:

6 $$\begin{aligned} K=\sqrt{N+M}+a \end{aligned}$$

in which *a* is a constant with an empirical value range of 0 to 10.

In order to improve the stability of model training, the data is first normalized before training the neural network model. This paper scales all sample data to the range of [0,1], which can be calculated by:

7 $$\begin{aligned} x^n_i=\frac{x_i-x_{min}}{x_{max}-x_{min}} \end{aligned}$$

where $x^n_i$ is the normalized data, $x_i$ is the orignal data, $x_{max}$ and $x_{min}$ represent the maximum and minimum values of the original data $x_i$, respectively.

In the BP-NN, we use $\omega _{n,k}^{i}$ and $b_{k}^{i}$ to represent the weights and thresholds from the input layer to the hidden layer, respectively. And $\omega _{k,m}^{o}$ and $b_{m}^{o}$ to represent the weights and thresholds from hidden layer to output layer, respectively. n, k, m is the neuron number in the input layer, hidden layer and output layer, respectively. Then, the output value of the *k*-th neuron ($1 \le k \le K$) in the hidden layer $net_{k}^{i}$ can be calculated by:

8 $$\begin{aligned} net_{k}^{i}=\sum \limits _{n=1}^{N}{\omega _{n,k}^{i}}{{s}_{n}}-b_{k}^{i} \end{aligned}$$

In order to enhance its ability to learn complex nonlinear relationship, activation function is adopted in the output of the hidden layer. Here, ReLU function was selected as the activation function, and it is defined as:

9 $$\begin{aligned} f(x)=max(0,x) \end{aligned}$$

At this time, the output value of the *k*-th neuron in the hidden layer through the activation function $net_{k}^{h}$ can be written as:

10 $$\begin{aligned} net_{k}^{h}=f\left( \sum \limits _{n=1}^{N}{\omega _{n,k}^{i}}{{s}_{n}}-b_{k}^{i} \right) \end{aligned}$$

The output value of the *m*-th ($1 \le m \le M$) neuron in the output layer $net_{m}^{o}$ can be calculated by:

11 $$\begin{aligned} net_{m}^{o}=\sum \limits _{k=1}^{K}{\omega _{k,m}^{o}}net_{k}^{h}-b_{m}^{o} \end{aligned}$$

According to Equation 10 and 11, the data mapping relationship between the system state *S* and alignment state $\Gamma$ can be calculated by:

12 $$\begin{aligned} \tau _m = net_{m}^{o}=\sum \limits _{k=1}^{K}{\omega _{k,m}^{o}}net_{k}^{i}-b_{m}^{o} =\sum \limits _{k=1}^{K}{\left( \omega _{k,m}^{o}f(\sum \limits _{n=1}^{N}{\omega _{n,k}^{i}}{{s}_{n}}-b_{k}^{i})-b_{m}^{o} \right) } \end{aligned}$$

In the BP-NN, if the predition error does not meet the requirements, it will back propagate the error from output layer to input layer. Here, gradient descent method was used to update the weights and thresholds. The prediction error between the predicted value and the actual value is defined as:

13 $$\begin{aligned} E=\frac{1}{m}\sum \limits _{i=1}^{m}{{{({{y}_{i}}'-{{y}_{i}})}^{2}}} \end{aligned}$$

where $${y_i}'$$ is the predicted value and $$y_i$$ is the actual value. And the weights *w* $$(w_{n,k}^{{i}}$$ or $$w_{k,m}^{{o}})$$ and thresholds *b* ($$b_{k}^{{i}}$$ or $$b_{m}^{{o}}$$) in BP-NN will be updated by:

14 $$\begin{aligned} & w=w-\alpha \frac{dE}{dw} \end{aligned}$$

15 $$\begin{aligned} & b=b-\alpha \frac{dE}{db} \end{aligned}$$

where $\alpha$ is the learning rate. After multiple rounds of learning and training, the prediction error will meet the accuracy requirements. Then, the model can be deployed and applied to help solve the optimization problem as shown in Equation 4.

### The heuristic optimization algorithm

To solve the alignment control problem formulated in Equation 4 and try to obtain the global optimal solution, we proposed a heuristic algorithm based on the genetic algorithm. The main steps of the algorithm are as follows:

Step 1: Encoding and initialization. We use binary number to represent the pressures and encode them into an array. Each pressure can be seen as a gene and each array can be seen as a chromosome. A population is composed of several chromosomes. For a pressure, the encoding length can be calculated by:

16 $$\begin{aligned} L_{gene}^{i}=\lceil {{\log }_{2}}(\frac{p_{i}^{u}-p_{i}^{l}}{\psi }+1) \rceil \end{aligned}$$

where $\psi$ means the resolution of the pressure and $\lceil \centerdot \rceil$ represents rounding up. For example, the resolution of the pressure is 0.01Mpa, and the upper bound $p_i^u$ and the lower bound $p_i^l$ is 2.0 and 0, respectively. Then, $L_{gene}^{i}=\lceil {{\log }_{2}}(\frac{2-0}{0.01}+1) \rceil =\left \lceil 7.65 \right\rceil =8$. Without loss of generality, we can assume that the safe constraints of all air springs are the same. Let ${{L}_{gene}}=L_{gene}^{i}$, then the chromosome length can be calculated by:

17 $$\begin{aligned} {{L}_{chrome}}=\sum \limits _{i=0}^{{{N}_{P}}}{L_{gene}^{i}}={{N}_{P}}\cdot {{L}_{gene}} \end{aligned}$$

Besides, we also need to initialize the population size $N_{pop}$, crossover probability and mutation probability. And randomly generate $N_{pop}$ chromosomes to form an initialized population.

Step 2: Calculate the fitness value. The fitness value is used to evaluate the quality of a chromosome. And the larger the fitness value, the better the chromosome quality. Considering that the control objective is to minimize *d*(*S*), the fitness value can be calculated by:

18 $$\begin{aligned} F=\frac{\text {1}}{d(S)}\text {=}\frac{1}{\big | f(S) \big |+\xi \centerdot \sqrt{\sum \limits _{i=1}^{N}{{{({{p}_{i}}-\overset{\_}{\mathop {p}}\,)}^{2}}}}} \end{aligned}$$

Step 3: Selection operation. Since the good chromosome has large fitness value, we use the proportionate selection method to select the chromosome. The probability of each chromosome being selected can be calculated as:

19 $$\begin{aligned} prob^s_i=\frac{F_i}{\sum \nolimits _{j=1}^{N_{pop}}{{F_j}}} \end{aligned}$$

where $F_i$ is the fitness value of *i*-th chromosome in the population. Besides, to ensure the convergence of the algorithm, we keep the best chromosome in the population^22^.

Step 4: Crossover operation. Crossover simulates population reproduction and can increase population diversity. The new chromosomes are formed by exchanging genes at the same position between different chromosomes. For two chromosomes, the probability of each gene exchanging with each other is $prob_c$, that is:

20 $$\begin{aligned} \left\{ \begin{aligned} g_{ik} = g_{jk}&\\ g_{jk} = g_{ik}&\end{aligned} \right. \end{aligned}$$

where $g_{ik}$ and $g_{jk}$ is the *k*-th gene for *i*-th and *j*-th chromosome, respectively.

Step 5: Mutation operation. In order to enrich population diversity and quickly converge to the optimal solution, mutation is introduced in the GA. Here, the probability of variation is denoted by $prob_m$. Each bit of a gene is selected for mutation with the probability $prob_m$, the value after mutation is updated by:

21 $$\begin{aligned} {{b}_{ik}^j}=({{b}_{ik}^j}+1) \bmod 2 \end{aligned}$$

where ${b}_{ik}^j$ is the *j*-th binary bit in *k*-th gene for *i*-th chromosome.

Step 6: Generate new population and calculate the fitness values. If it does not meet the requirements, retrun to Step 3; otherwise, the evolution is complete, and the best chromosome will be selected and decoded into an array of optimal air spring pressures, denoted by $P^{opt}=\{p^{opt}_1,...,p^{opt}_{N_P}\}$.

Finnaly, the optimized air spring pressures will be transmitted to the controller to ajust the shaft alignment state.

### The design of soft-constraint PID controller

According to the optimal air spring pressures generated by the heuristic algorithm, we can adjust the air spring pressures by controlling the solenoid valve. To calculate the inflation or deflation time for an air spring with the solenoid valve, we need to know the flow rate Q and pressure change rate. The flow rate *Q* through the solenoid valve can be expressed as:

22 $$\begin{aligned} Q = C_d A \sqrt{\frac{2 \Delta p}{\rho }} \end{aligned}$$

where $C_d$ is the flow coefficient of the valve, *A* is the effective area of the valve, $\Delta p$ is the pressure difference across the valve, $\rho$ is the density of the gas. The rate of change of pressure $\frac{dp}{dt}$ in the air spring can be described by:

23 $$\begin{aligned} \frac{dp}{dt} = \frac{Q}{V} \end{aligned}$$

where *V* is the volume of the air spring. To calculate the operating time $T_{p_i \rightarrow p_i^{opt}}$ required to change the pressure from an initial pressure $p_i$ to the optimized pressure $p_i^{opt}$, we can integrate the pressure change over time:

24 $$\begin{aligned} T_{p_i \rightarrow p_i^{opt}} = \int _{p_i}^{p_i^{opt}} \frac{dp}{\frac{Q}{V}} \end{aligned}$$

In an ideal situation, when the pressure difference between the two ends of the solenoid valve is fixed, the flow rate *Q* of the solenoid valve is constant. The operating time of the sole valves can be directly calculated according to Eqs.24. However, in practical engineering, these parameters are difficult to measure. The PID controller has been applied in a wide range of control applications for its simplicity, adaptability, and proven effectiveness. It is particularly suitable for systems where the exact mathematical model is either unknown or difficult to obtain. Therefore, we introduce the PID control algorithm in this paper.

In order to achieve accurate and stable air pressure control, we designed a soft constrained PID controller to generate specific control strategies, that is, to convert the difference between current air spring pressures and the optimal pressures into the operating time of inflation or deflation solenoid valves. The details of the soft constraint PID control algorithm is shown in Algorithm 1:

**Algorithm 1:**
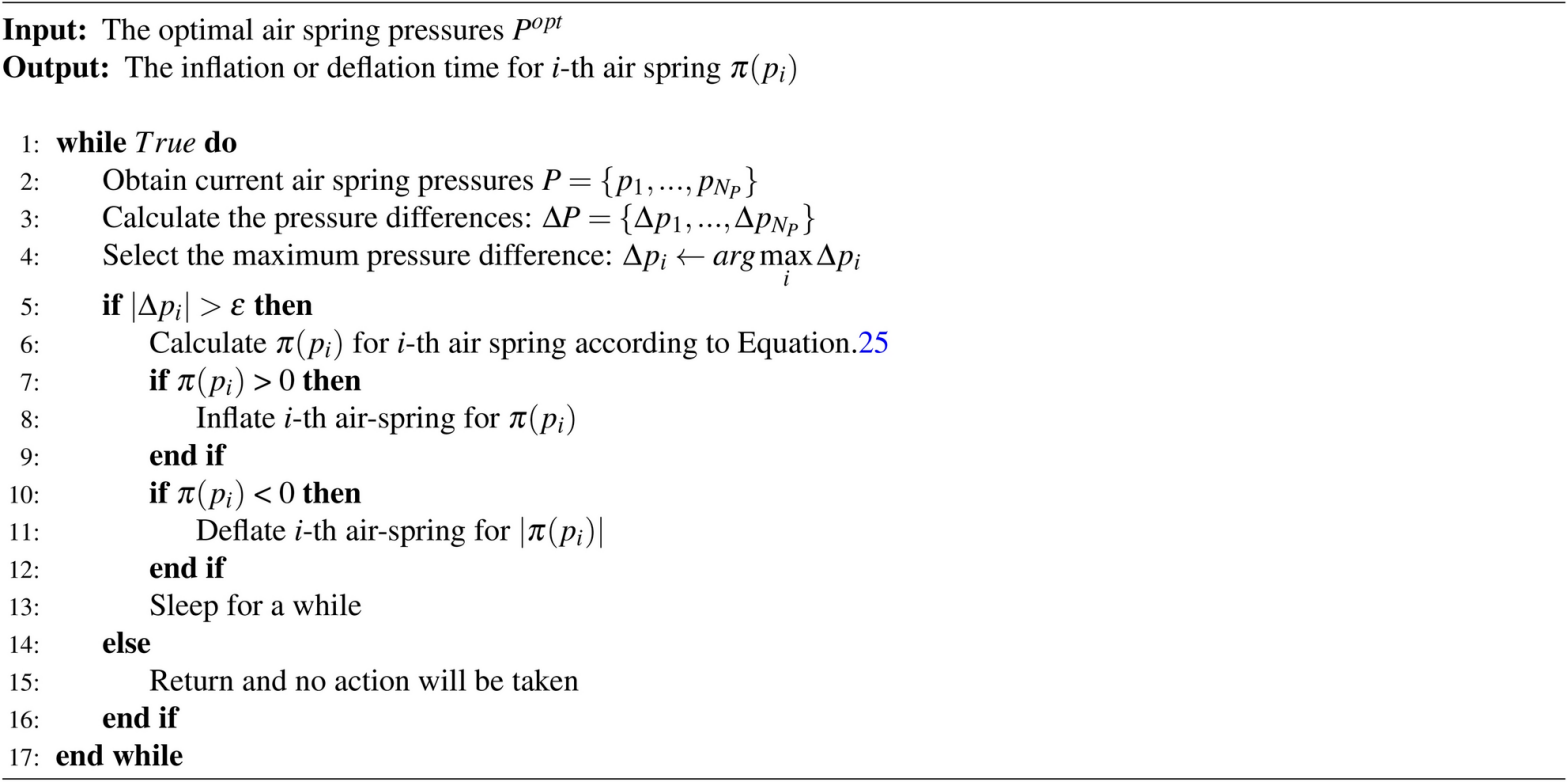
The soft constraint PID control algorithm

In the process of designing a controller, three aspects need to be considered: 1) accuracy. Accurate control of air spring pressure is necessary to achieve precise shaft alignment control. And the operating time $\pi (p_i)$ of inflation or deflation *i*-th air spring can be calculated by:

25 $$\begin{aligned} \pi ({{p}_{i}})={{k}_{p}}\cdot \Delta p_i+{{k}_{i}}\cdot \int \limits _{0}^{t}{\Delta p_i}dt+{{k}_{d}}\cdot \frac{d\Delta p_i}{dt} \end{aligned}$$

where the three parameters $k_p,k_i,k_d$ are all non-negative, controlling the proportional, integral and differential part, respectively. In a practical system, considering that it is hard to calculate the differential item, we use a pseudo-differential method. The differential part in Eqs.25 is calculated by:

26 $$\begin{aligned} \frac{d\Delta {{p}_{i}}}{dt}=\frac{\Delta {{p}_{i}}-\Delta p_{i}^{last}}{\pi {{({{p}_{i}})}^{last}}} \end{aligned}$$

where $\Delta p_{i}^{last}$ means the pressure difference in last control process and $\pi {{({{p}_{i}})}^{last}}$ means the operating time in the last control process. If it is the first time to calculate the operating time and there is no record for $\Delta p_{i}^{last}$ and $\pi {{({{p}_{i}})}^{last}}$, the differential part is set to 0. And $\Delta p_i=p_i^{opt}-p_i, 1 \le i \le N_P$. When $\Delta p_i>0$, $\pi (p_i)>0$ and it means the air spring need inflation operation; when $\Delta p_i<0$, $\pi (p_i)<0$ and it means air spring need deflation operation, and the operating time is $|\pi (p_i)|$; otherwise, no operation will be taken.

2) Stability. Considering the measurement and disturbance errors in air spring pressure, the controller allows for a certain degree of error to avoid unnecessary action of the solenoid valve. Assuming the allowable error is $\varepsilon$, when $|\Delta p_i| < \varepsilon$, it is considered that the optimal pressure has been reached and no control will be performed. In engineering, the setting of the soft constraint value should fully consider factors such as the rated working pressure of the air spring, as well as the measurement accuracy and measurement error of the pressure sensor. If the soft constraint is set too large, it will affect the accuracy of alignment control.

3) Time delay. After the air spring is inflated, the air pressure will first increase, and then the air spring height may increase, resulting in a decrease of the pressure. Also, the pressure change of one air spring may cause other air spring pressures to change accordingly. Thus, after the inflation and deflation operation, the system requires a certain amount of time to stabilize, exhibiting time delay. Therefore, we sort the pressure difference first and prioritize controlling the air spring corresponding to the maximum pressure difference.

## Performance evaluation

In this section, experiments are conducted to verify the availability of the proposed digital-twin driven alignment control method. Considering that the modeling, optimization and control are decoupled in the proposed method, we first evaluate the precision of the digital-twin prediction model using different metrics. Then, the heuristic algorithm based on GA is tested and key parameters in it are discussed as well. According to the optimized air spring pressures, the effectiveness of PID controller is validated with an actual ASVIS for main engine.

### Experiment setup

**Fig. 3:**
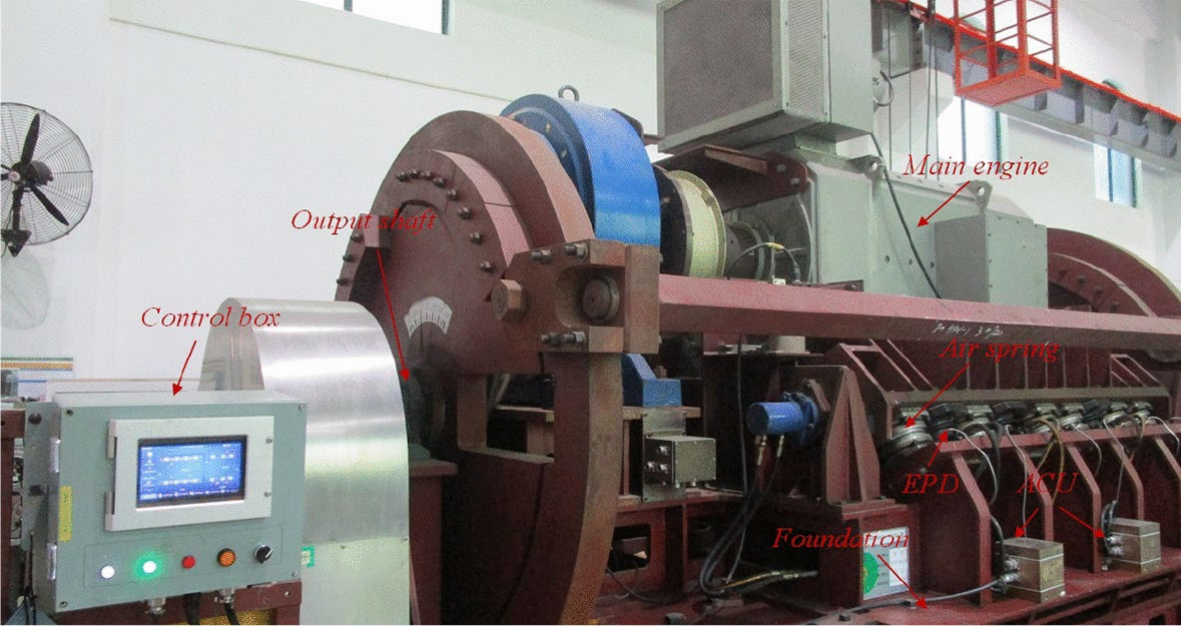
The physical entity of ASVIS used in the experiment.

The experiments are carried out with an ASVIS for main engine, as shown in Figure 3. In the ASVIS, 8 air springs are used to support the main engine with an inclined angle of $30^{\circ }$. Each air spring is connected with an air-control-unit (ACU), which has sensors to monitor the air spring’s pressure and has solenoid valves to perform inflation and deflation. 7 displacement sensors are deployed to measure and calculate the alignment state. All these devices are connected to a control box through an industrial bus, and the monitored data are recorded and stored in it. The control box can communicate with other high-performance servers by Ethernet. Besides, there is an emergency protection device (EPD) for the ASVIS to limit excessive displacement in emergencies. When the operating condition changes too drastically and the system detects a drastic change in the alignment state, the ASVIS will start EPD, and it can be seen as a group of rigid supports to fix the bearing equipment, so as to protect the safe operation of the equipment.

In the experiment, the prediction model training and optimization calculation is implemented in a server, in which the GPU is NVIDIA GeForce RTX 3070 and CUDA version is 12.4.89. And the air spring control is performed in the control box. The prediction model and optimization algorithm are developed by Python (version 3.12.2), and the library pytorch (version 2.2.1) is used to establish the neural network. For BP-NN, the learning rate is set to 0.02, the nodes number in hidden layer is set to 10, and Adam optimizer is applied in the back-propagation algorithm. Three typical metrics are used to evaluate the prediction performance, that is, mean square error (MSE), mean absolute error (MAE), and determinant coefficient ($R^2$). MSE can be calculated by Equation.13. The mean absolute value is defined as:

27 $$\begin{aligned} MAE=\frac{1}{n}\sum \limits _{i=1}^{n}{\left| {{y}_{i}}'-{{y}_{i}} \right| } \end{aligned}$$

where n is the amount of collected data, ${{y}_{i}}'$ is the *i*-th predicted value and $y_i$ is the *i*-th actural value. The determinant coefficient $R^2$ is defined as:

28 $$\begin{aligned} {{R}^{2}}=1-\frac{SSE}{SST}=\frac{SSR}{SST} \end{aligned}$$

in which $SST=\sum \nolimits _{i=1}^{n}{(y_i-{\bar{y}}\,)^2}$ is the total sum of squares, $SSR=\sum \nolimits _{i=1}^{n}({y_i}'-{\bar{y}}\,)^2$ is the regression sum of squares and $SSE=\sum \nolimits _{i=1}^{n}{{(y_i-{y_i}')}^2}$ is the residual sum of squares. ${\bar{y}}$ is the average value of $y_i$.

In order to verify the effectiveness of the prediction model, we have collected about 20000 groups data in the real ASVIS as shown in Figure 3. Considering that we mainly focus on how to control alignment state by adjusting air spring pressures in the paper, to simplify the model without losing generality, we assume that the operating condition remain unchanged. And each group of the data includes 8 air spring pressure values and alignment state values. We also randomly divide the data into training set (accounting for $80\%$) and test set (accounting for $20\%$). And the prediction model will be trained with the training set and tested with the test set.

For the heuristic algorithm, the multi-objective coefficient is set to $\xi =3$. We hope the vertical/horizontal offsets are less than 0.3mm and the vertical/horizontal angularities are less than 0.3mm/m. The safe pressure contraints for air springs are $0 \sim 2.0$. Thus, the constraints for the opimization problem are set to $\tau ^l_i=-0.3$, $\tau ^u_i=0.3$, $p^l_i=0$, $p^u_i=2.0$. In GA, the default population size is set to $N_{pop}=50$, the crossover probability and mutation probability is set to $prob_c=0.9$ and $prob_m=0.005$, separately. For PID controller, we have tuned the PID parameters to achieve a balance between accuracy and stability by evaluation, and they are set to $k_p=400$, $k_i=20$ and $k_d=1$. The soft-constraint $\epsilon =0.02$Mpa. And the unit of the operation time is milliseconds (*ms*).

### Results

**Fig. 4:**
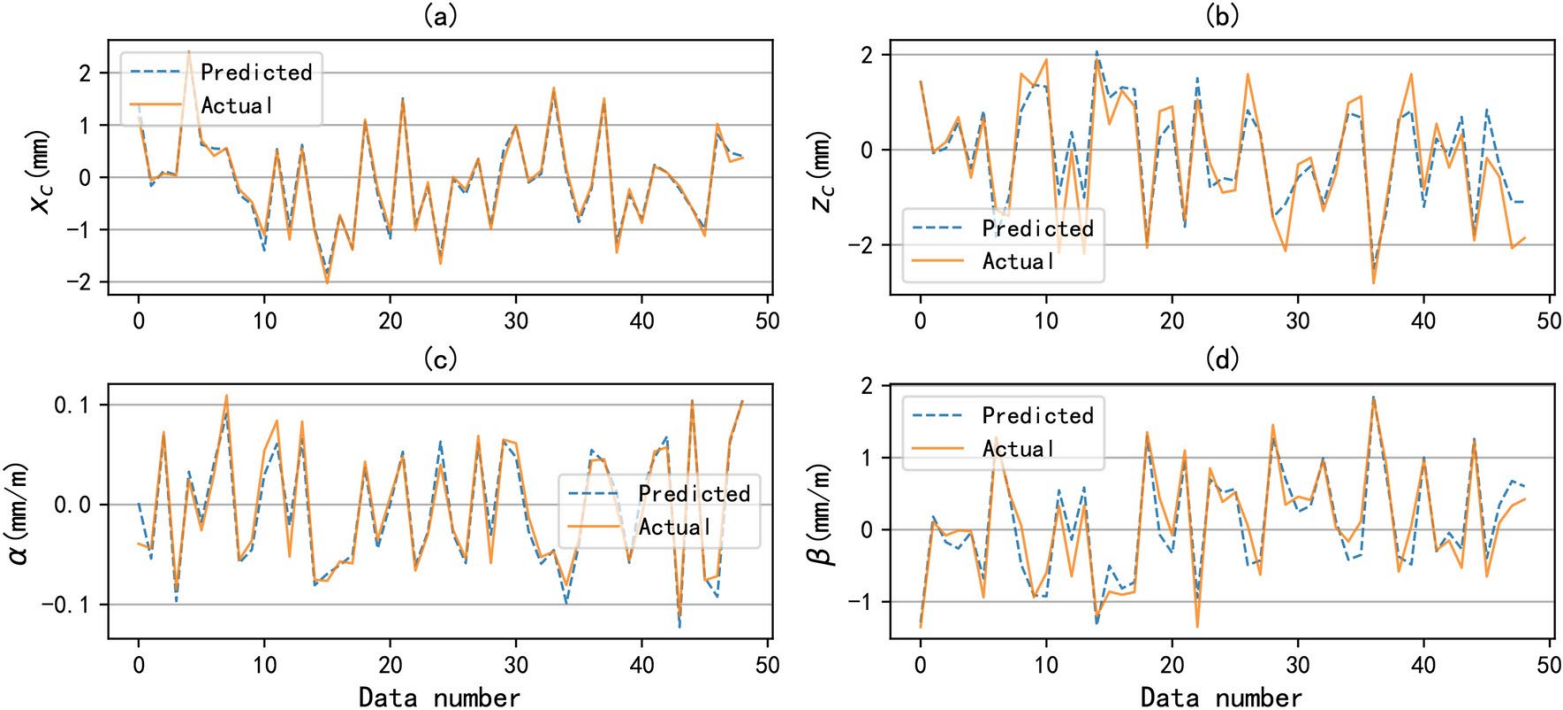
The comparison of the predicted values with prediction model and actual values. (**a**) horizontal offset; (**b**) vertical offset; (**c**) horizontal angularity; (**d**) vertical angularity.

1) To evaluate the effectiveness of the digital twin driven alignment control method, we first tested the precision of the prediction model. In order to visually demonstrate the predicted results, we randomly selected 50 data samples from the test set for display. Figures 4 (a), (b), (c), and (d) show the comparison between the predicted and actual values of the four components of the alignment state, namely horizontal offset $x_c$, vertical offset $z_c$, horizontal angularity $\alpha$, and vertical angularity $\beta$, respectively. It can be seen that the prediction model has good predictive performance in all four components. In order to fully evaluate the performance of the prediction model, we tested the model using different metrics, including MSE, MAE, and R2. Table 1 shows the evaluation of prediction results with different metrics for the alignment state vector and its four components, and these results are the average of 20 randomized trials. It can be seen that the MSE value of the alignment state vector is about 0.02, the MAE value is about 0.08, and the coefficient of determination is about 0.89. We can say that the prediction model performs well under different metrics. For single component, alpha performs the best on MSE and MAE metrics, but R2 performs the worst because the value of a itself changes less. In summary, the test results indicate that the predictive model can accurately describe the data mapping relationship between air spring pressures and alignment state.

**Table 1:** The results of the digtal-twin prediction model with different metrics.

**Metrics**	$x_c$	$z_c$	$\alpha$	$\beta$	Total
MSE	0.0103	0.0536	0.0003	0.0168	0.0203
MAE	0.0783	0.1574	0.011	0.0888	0.0839
R2	0.9884	0.9557	0.9415	0.9716	0.8918

**Fig. 5:**
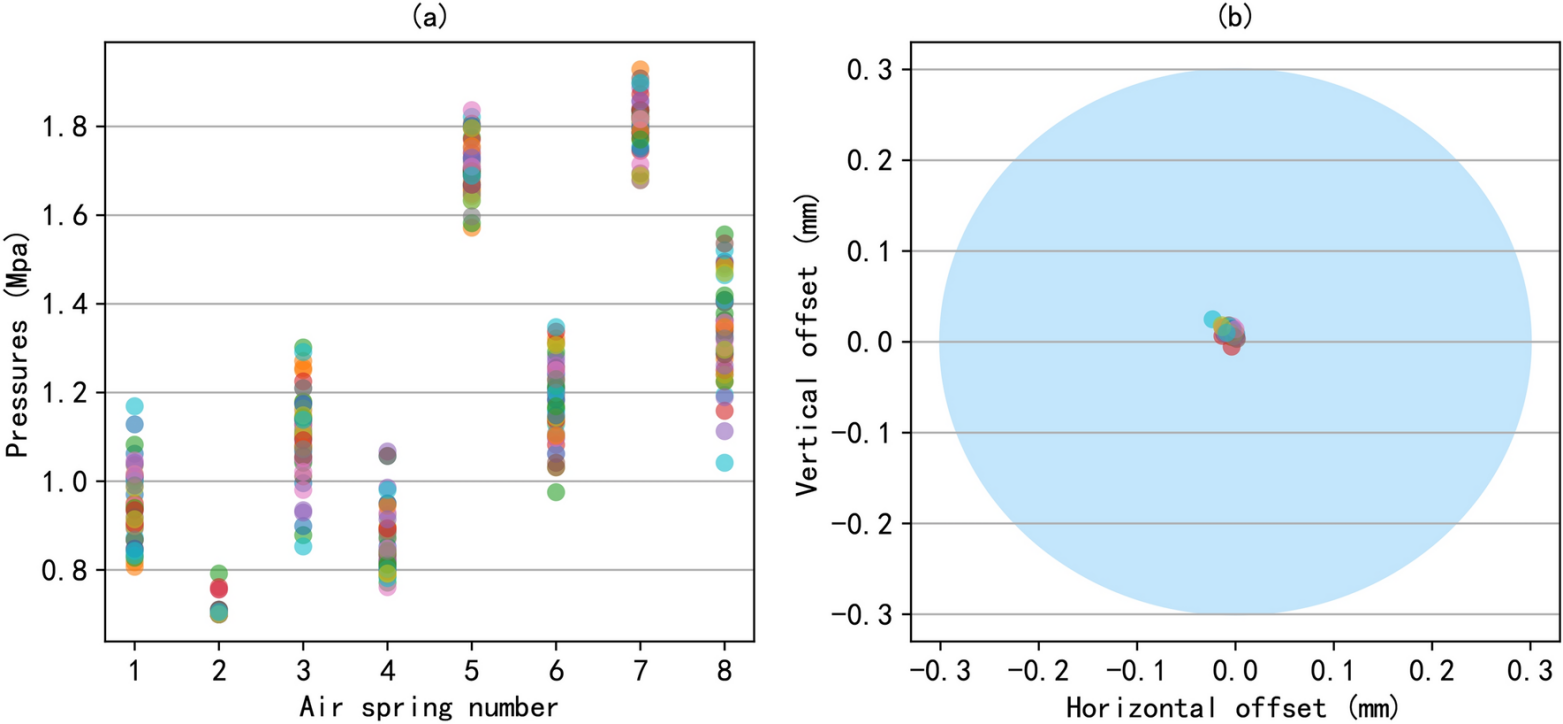
The optimization results with 50 sets of random initialization algorithm parameters. (**a**) the 50 sets of air spring pressures distribution; (**b**) the 50 sets of alignment state.

2) To verify the effectiveness of the optimization algorithm, based on the digital twin model, we use the proposed heuristic algorithm to calculate the optimal air spring pressures and corresponding alignment state. Here, 50 sets of random initialization algorithm parameters were tested, and 50 sets of air spring pressure values and corresponding alignment states were obtained. Figure 5(a) shows the 50 sets air spring pressure distribution, and it can be seen that the optimal air spring pressures is not unique. This also provides the possibility for multi-objective optimization. Figure 5(b) shows the alignment states corresponding to the 50 sets of optimal air spring pressures, with the shaded areas indicating the alignment control target. It can be seen that the optimized pressures are all within the safety constraint range and the system can achieve a high degree of alignment accuracy.

**Fig. 6:**
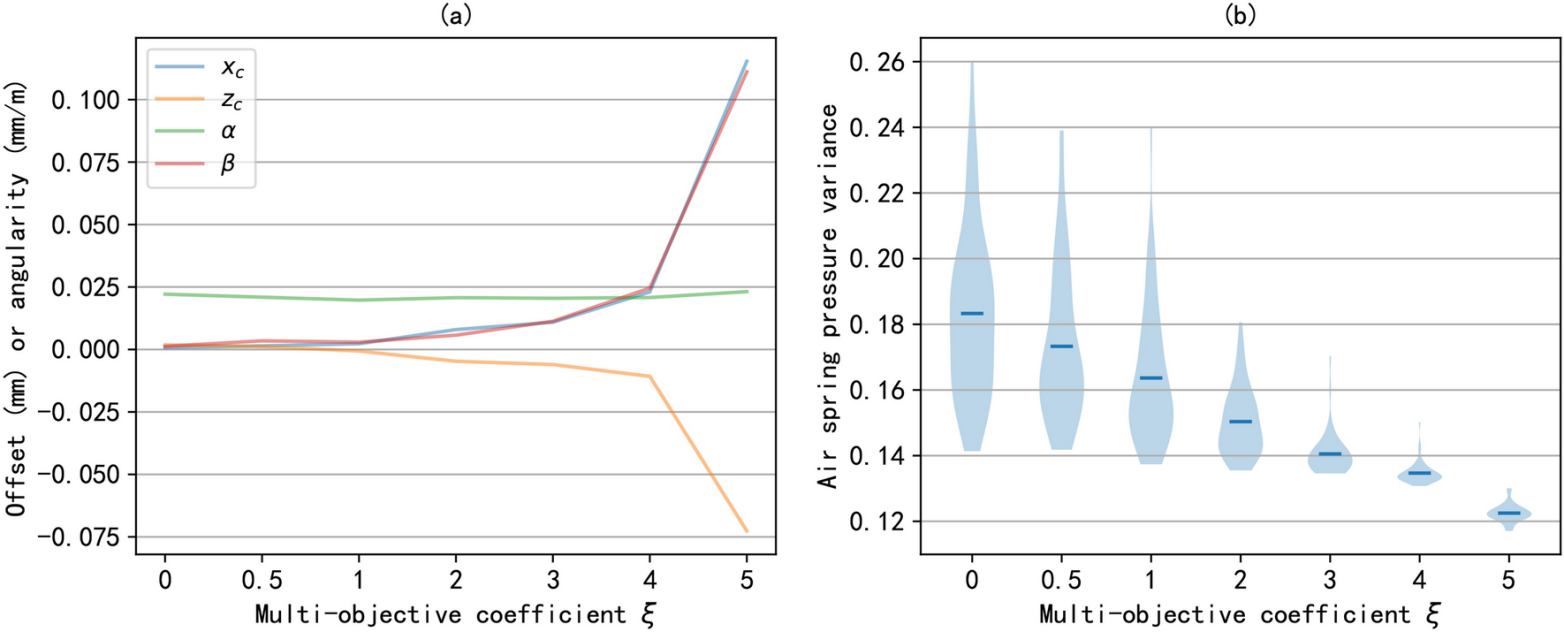
The optimization results with different multi-objective coefficients. (**a**) the performance of alignment state; (**b**) the air spring pressure variance.

Then, we studied the impact of the multi-objective coefficient $\xi$ on the optimization results. This paper transforms multi-objective optimization problems into single objective optimization problems through multi-objective coefficients. Therefore, the value of the multi-objective coefficient will directly affect the optimization results. Figure 6 shows the performance of alignment state and air spring pressure variance under different multi-objective coefficients. Specifically, Figure 6(a) shows the performance of the four components of the alignment state under different multi-objective coefficients. It can be seen that as the multi-objective coefficient value increases, the optimized alignment state will gradually deteriorate, as the optimization objective will be more inclined to reduce the variance of air spring pressure. Figure 6(b) shows the variance of a set of air spring pressures under different multi-objective coefficients. It can be seen that as the coefficient value increases, the variance of air spring pressure decreases, and the load is more evenly distributed on different air springs. According to Figures 6(a) and 6(b), when the multi-objective coefficient is $\xi =3$, we can achieve good alignment while maintaining load balance.

**Fig. 7:**
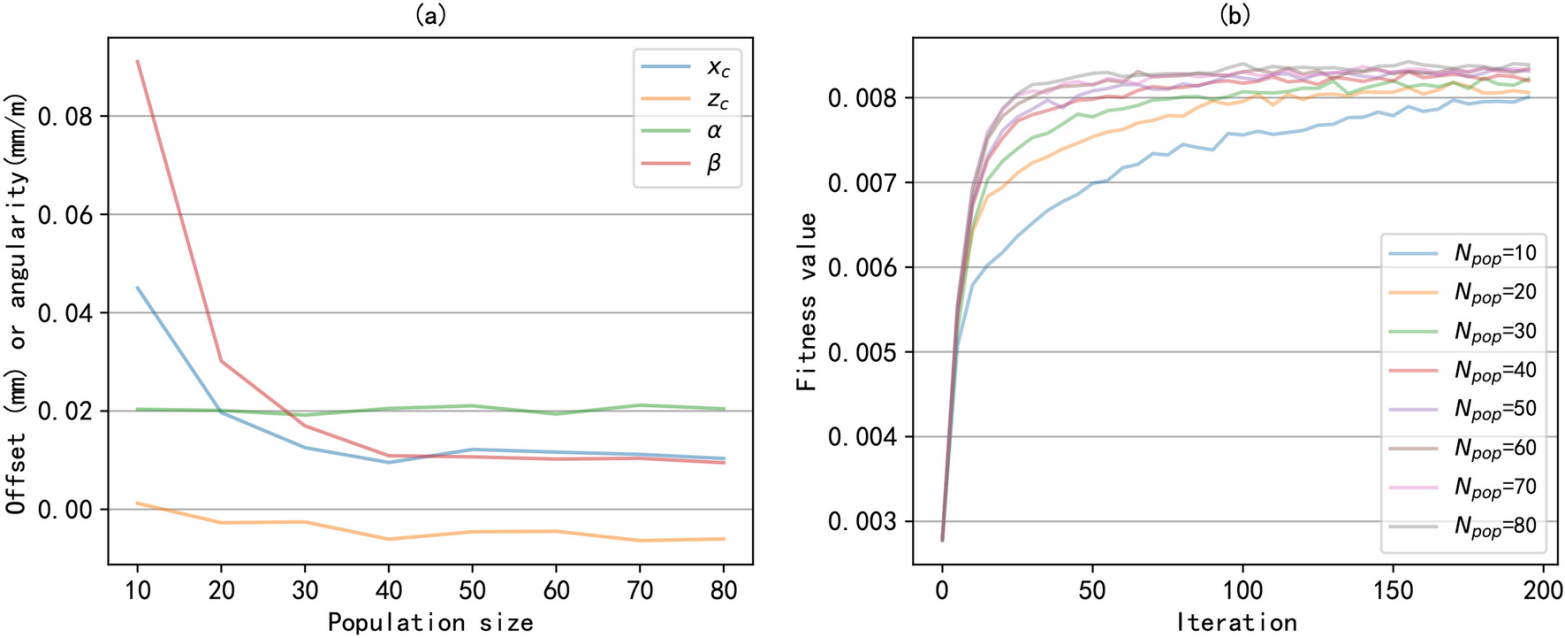
The optimization results with different population size. (**a**) the optimal alignment state; (**b**) the fitness values during the training process.

**Fig. 8:**
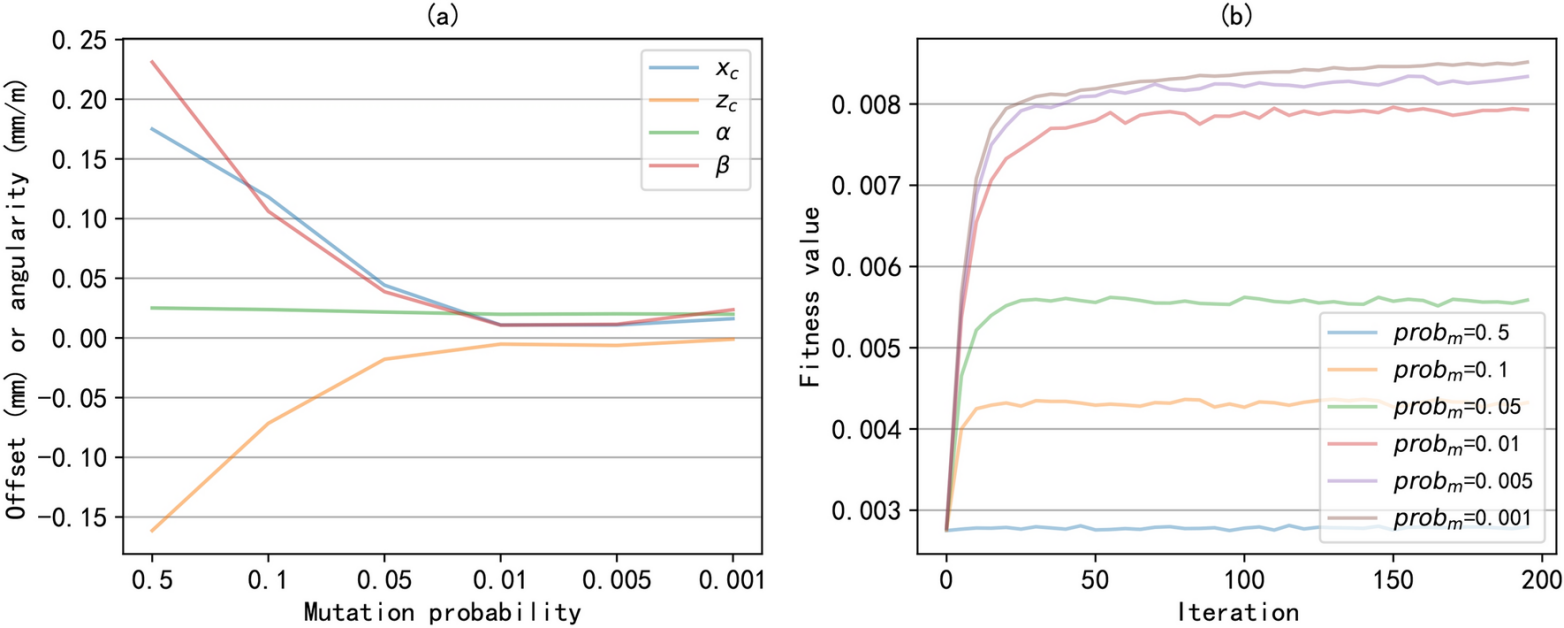
The optimization results with different mutation probability. (**a**) the optimal alignment state; (**b**) the fitness values during the training process.

In addition, we investigated the influence of the important parameters in genetic algorithms on optimization results, namely population size and mutation probability. The population size may affect the convergence speed of the algorithm. Figure 7 shows the optimal alignment state under different population sizes and the fitness values during the training process. From Figure 7(a), it can be seen that when the population size is too small, it is difficult to converge and obtain the optimal alignment state, resulting in a large alignment control error. As shown in Figure 7(b), increasing the population size is beneficial for improving the convergence speed of the algorithm. Of course, an increase in population size will increase computing and storage resources. And when the population size reaches a certain scale, increasing the population size has limited effect on improving the accuracy of optimization results. In this experiment, the optimal alignment state can be obtained when the population size is 40. Mutation is mainly aimed at improving population diversity and enhancing the efficiency of global optimization. Figure 8 shows the optimal alignment states obtained under different mutation probabilities and the convergence of the algorithm during the training process. we can see that when the mutation probability is too high, it will result in a deterioration of the optimal alignment state. From Figure 8(b), it can be seen that the convergence speed of different mutation probabilities is basically the same. However, excessive mutation probability can lead to a decrease in fitness value, that is, a decrease in the optimal alignment state, consistent with the results in 8(a). In this experiment, a mutation probability of 0.01 can achieve good optimization results.

**Fig. 9:**
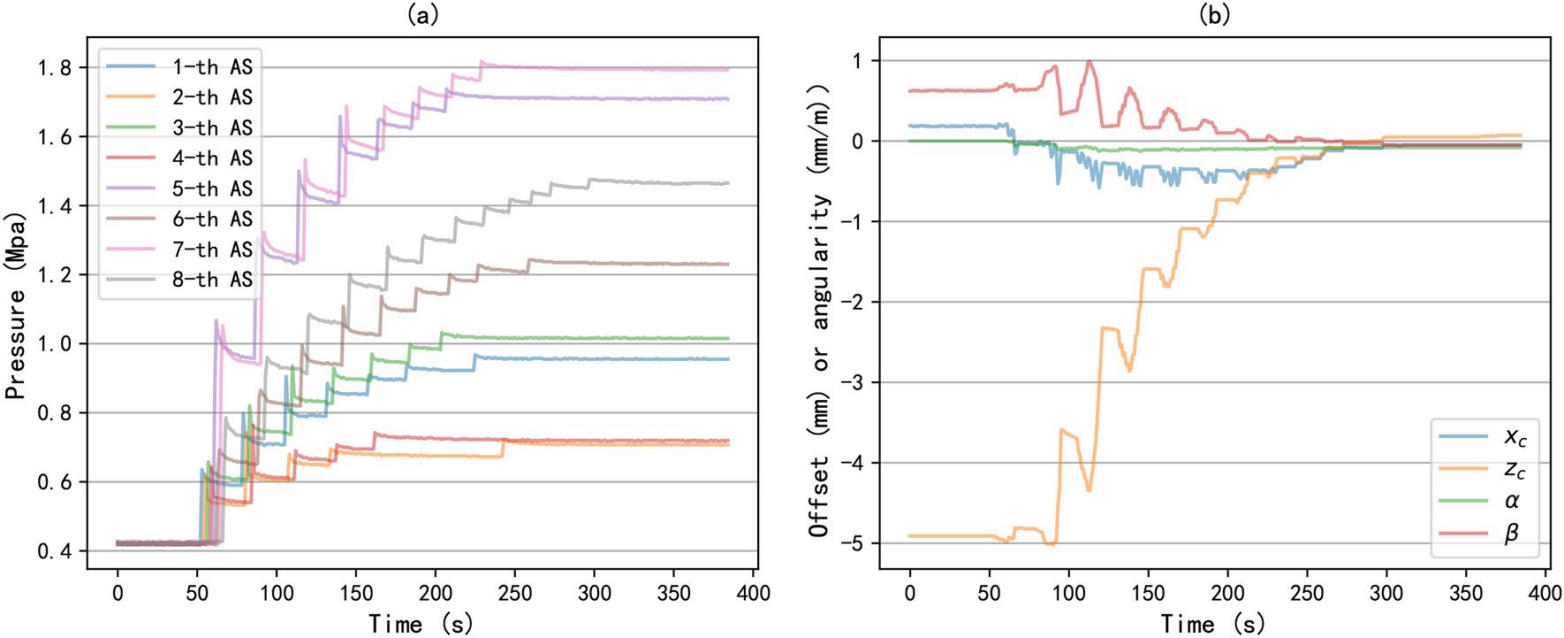
The alignment control process of the ASVIS. (**a**) the change of the air spring pressures; (**b**) the change of the alignment state.

3) To verify the effectiveness of the PID controller, we initialize the air spring pressures to 0.4Mpa, and let the controller automatically control the air springs to complete the alignment control according to the optimized air spring pressures. We monitored and recorded the alignment control process, as shown in Figure 9. Figure 9(a) shows the change of air spring pressure during the control process, we can see that the air spring pressures will first rise and then decrease after they are inflated, which is caused by the need to rebalance the system after the pressure change of a single air spring. This phenomenon can also be understood as the delay characteristic of the system response. Accordingly, Figure 9(b) shows the change of alignment state of shafting in the control process, and it can be seen that the sudden change of pressure of a single air spring will also lead to a rapid change of alignment state. Thus, when setting PID parameters, it should be considered that the single control time should not be too large, otherwise, the system may jitter in a short time. In this experiment, after each control action is completed, the system will sleep for one second before performing the next control action. Finally, as the air spring pressures gradually approach the optimal pressures, the alignment state also converges to the optimal alignment state. The accuracy of the alignment state is within 0.1mm (vertical/horizontal offsets) and 0.1mm/m (vertical/horizontal angularities), while traditional methods maintain the alignment control accuracy at around 0.5 (mm or mm/m)^15,17^. Therefore, it can be said that the proposed controller can control the shafting alignment state by controlling the air spring pressures.

## Conclusions

This paper studies the marine shafting alignment control problem with air spring vibration isolation systems and proposes a digital twin-driven alignment control method. Firstly, a digital twin prediction model for the air spring isolation system was established based on BP neural network, accurately describing the mapping relationship between air spring pressures and alignment state. Then, the alignment control problem is transformed into a multi-objective nonlinear optimization problem, and a heuristic algorithm based on genetic algorithm is designed to optimize the pressure values corresponding to the optimal alignment state. In order to accurately control air spring pressures, we have also developed a soft constrained PID controller that can transform the pressure difference between current pressures and optimal pressures into specific control strategies. To validate the effectiveness of the proposed method, we conducted experiments in a real air spring vibration isolation system, and the experimental results showed that the proposed digital twin-driven alignment control method can accurately and effectively achieve the goal of precise shaft alignment control by optimizing and adjusting the air spring pressures.

## Data Availability

The datasets used and analysed during the current study available from the corresponding author on reasonable request.
